# Modeling Quantum Dot Systems as Random Geometric Graphs with Probability Amplitude-Based Weighted Links

**DOI:** 10.3390/nano11020375

**Published:** 2021-02-02

**Authors:** Lucas Cuadra, José Carlos Nieto-Borge

**Affiliations:** 1 Department of Signal Processing and Communications, University of Alcalá, 28801 Alcalá de Henares, Spain; 2 Department of Physics and Mathematics, University of Alcalá, 28801 Alcalá de Henares, Spain; josecarlos.nieto@uah.es

**Keywords:** quantum dot, disorder array of quantum dots, probability amplitude, complex networks, spatial network, Random Geometric Graphs, quantum transport

## Abstract

This paper focuses on modeling a disorder ensemble of quantum dots (QDs) as a special kind of Random Geometric Graphs (RGG) with weighted links. We compute any link weight as the overlap integral (or electron probability amplitude) between the QDs (=nodes) involved. This naturally leads to a weighted adjacency matrix, a Laplacian matrix, and a time evolution operator that have meaning in Quantum Mechanics. The model prohibits the existence of long-range links (shortcuts) between distant nodes because the electron cannot tunnel between two QDs that are too far away in the array. The spatial network generated by the proposed model captures inner properties of the QD system, which cannot be deduced from the simple interactions of their isolated components. It predicts the system quantum state, its time evolution, and the emergence of quantum transport when the network becomes connected.

## 1. Introduction

**Figure 1:**
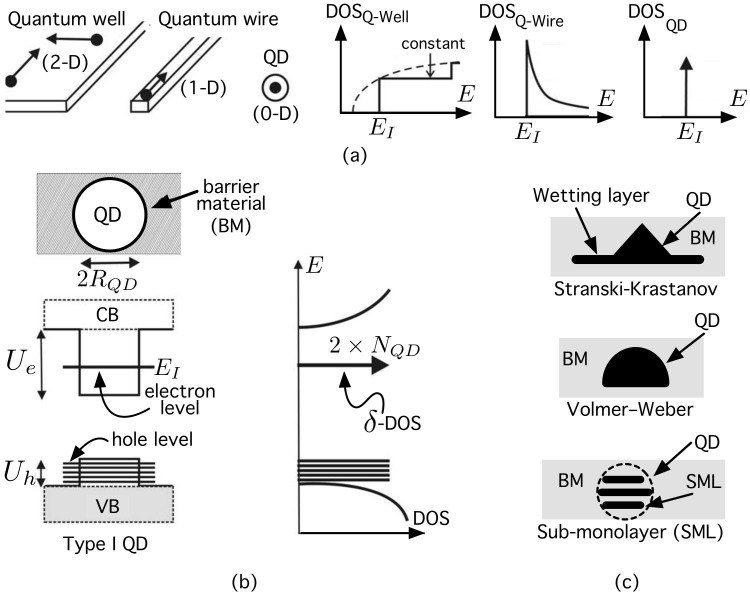
(**a**) Conceptual representation of nano-structures and their corresponding density of states (DOS) for quantum wells, quantum wires and quantum dots; (**b**) confinement potentials for electrons ($U_{e}$) and holes ($U_{h}$) in a type I QD (or simply, QD), and its corresponding density of states; (**c**) different classes of growth in self-assembled quantum dots. See the main text for details.

Conceptually, a quantum dot (QD) [1] is a “small” (less than the de Broglie wavelength), zero-dimensional (0D) nanostructure that confines carriers in all three directions in space [2,3,4], and exhibits a delta density of states (DOS), unlike those of quantum wells (2D nanostructure [5]) and quantum wires (1D) [2], as shown in Figure 1a. In particular, a (type I) semiconductor QD is a heterostructure [6] made up of a small island (generally 10–20 nm in size) of a semiconductor material (“dot material”) embedded inside another with a higher bandgap (“barrier material” –BM–), which is able to confine both electrons and holes, as illustrated in Figure 1b). This creates discrete energy levels for carriers, and modifies both its electronic and optical properties [3]. The problem of manufacturing high densities of high-quality QDs is successfully addressed by using self-assembled QD (SAQD) [7] technologies. Figure 1c shows different SAQD growth modes. In the Stranski–Krastanow (SK) growth mode [8], the deposition of the dot material starts with the formation of a two-dimensional, very thin wetting layer (WL), and when a critical amount of strained dot material has been deposited, the formation of pyramidal QDs occurs to relax strain. In the Volmer–Weber (VW) mode, the QDs grow directly on the bare substrate [9]. Sub-monolayer (SML)-QDs [10] can be formed as a disk or as a spherical QD because the growth method consists of multilayer deposition with a fraction of a monolayer of dot material on the barrier matrix [8]. SML-QDs have several advantages over SK-QDs, such as a smaller base diameter (5–10 nm), higher dot density (~$5\times 10^{11}$ ${\mathrm{cm}}^{-2}$), and better control of QD size [10,11].

SAQD technologies help both research in Quantum Mechanics (QM) and manufacture novel devices. On the one hand, SAQD technologies assists in manufacturing high quality QDs for studying QM and exploring novel effects in electronics, photonics, and spintronics. For instance, SAQD-based devices help achieve single-electron charge sensing [12], entanglement between spins and photons [13,14], single-photon sources [15], or single-spin [16], and help also the control of Cooper pair splitting [17], spin transport [18], spin–orbit interaction [19], *g*-factor [20], and Kondo effect [21]. On the other hand, SAQD technologies allow for manufacturing high density of QDs, which are crucial for implementing opto-electronic devices such as QD-based light-emitting diodes (LEDs) [22], QD-memories [4,23], QD-lasers [24,25,26,27], QD-infrared photodetectors [8,28,29], and QD-solar cells [30]. A key point in these devices is that the position of carrier level(s) can be tuned by controlling the dot size [2], and, this, by modifying the growth conditions [6,10,11,31].

In this work, we model a system formed by QDs as a special *graph* in the effort of exploring electron transport. Our approach could be considered as belonging to Complex Networks (CN) Science. CN have become a multidisciplinary research field [32,33,34] for studying systems with a huge amount of components that interact with each other. These range from artificial systems (such as power grids [35,36], or the Internet [37]) to natural systems (vascular networks [38], protein interactions [39], or metabolic networks [40]). More examples showing the feasibility of CN to study many other systems can be found in [32,41,42] and the references therein. All these very different systems have in common that all of them can be described in terms of a graph [32]: a set of entities called nodes (or vertices) that are connected to each other by means of links (or edges). A node represents an entity (generator/load in a power grid [36], or a species in an ecosystem [43]), which is connected with others (linked by electrical cables in power grids, or related by trophic relationships in ecosystems). This way, any system can be encoded as a graph, a mathematical abstraction of the relationships (links) between the constituent units (nodes) of the system. In broad sense, CN science models not only the structure (topology), but also some dynamic phenomena such as information spreading [44], epidemic processes (both biological [45], and artificial viruses [46]) or cascading failures [47,48]. These are very common in large engineered networks: wireless sensor networks [49], Internet [50], power grids [35,51,52], or transportation networks [53]. Some of these CN, such as transportation networks, are networks in which the nodes are spatially embedded [54] or constrained to locations in a physical space with a metric. For many practical applications, the space is the two-dimensional space and the metric is the Euclidean distance $d_{E}$. This geometric constraint usually makes the probability of having a link between two nodes decrease with the Euclidean distance [53]. This particular subset of networks are called spatial networks (SN) [53,55] or spatially embedded CN [56]. A particular class of SN are Random Geometric Graphs (RGGs) [57] in which *N* nodes are uniformly distributed over the unit square, while the link between two nodes *i* and *j* is formed if the Euclidean distance $d_{E}\left(i,j\right)<r$, a given model parameter. RGGs have been successfully used to model wireless sensor networks [58] and ad hoc networks [59] in which *r* is related to the range of the radio devices.

The previous paragraph has shown that CN science has been applied to a broad variety of macroscopic, “classical” (non-quantum) systems. As will be shown in our review of the current state of the art in Section 2, CN science has also been used to study quantum nanosystems, although to a much lesser extent and not with the RGG approach that we will show throughout this paper.

**Figure 2:**
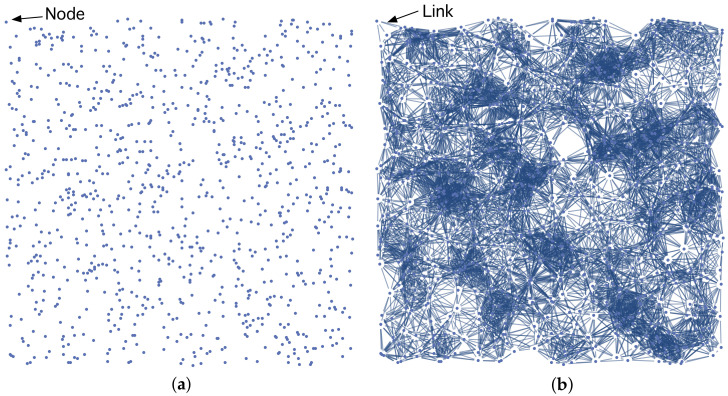
(**a**) Random distribution of QDs in $R^{2}$. Each QD, represented by a node, causes a quantum confinement potential. (**b**) A link between two nodes is allowed if and only if the electron wave-functions at such nodes have non-zero overlap. Any link has a weight that is quantified by such overlap. See the main text for details.

The purpose of our work is to propose a particular class of RGG whose links have weights computed according to QM with the aim of studying electron transport in a system of disordered QDs like the one in Figure 2a. It consists of a number of *N* QDs—for instance, a layer of SAQDs [6], which produce finite quantum confinement potentials (QCPs), randomly distributed in the physical metric space, $R^{2}$, characterized by the Euclidean distance, $d_{E}$.

**Figure 3:**
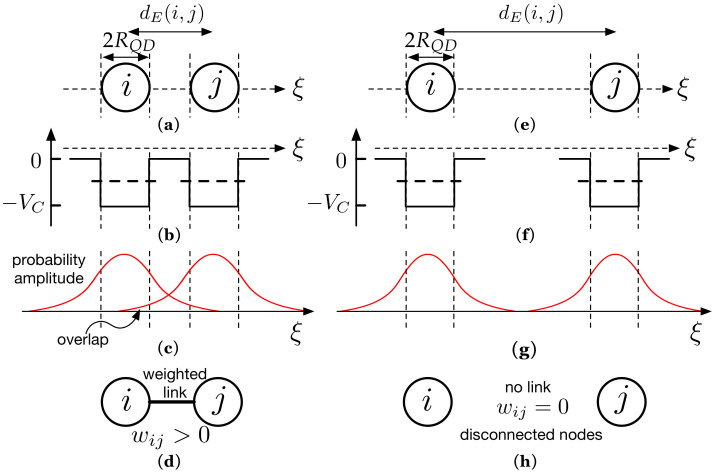
(**a**) Two of the QDs as those in Figure 2a. We consider QDs with radius $R_{QD}$ and whose centers are separated by a Euclidean distance $d_{E}\left(i,j\right)$. ξ represents an axis that passes through the center of both QDs. Along the ξ-axis, we have represented in (**b**) the corresponding QCP ($-V_{C}$ inside the node, 0 otherwise) and the electron probability amplitudes (**c**). An electron can be in both QDs with the represented probability amplitude. (**d**) We model this with a link whose weight $w_{ij}$ is given by the overlap. (**e**) Opposite case in which the QDs are so far apart (**f**) that there is no overlap (**g**) and link formation is not allowed (**h**).

In our model, any QD causing a QCP in Figure 2a is represented by a node. To understand how links have been generated in Figure 2b, it is convenient to have a look at Figure 3. On its left side, we have represented, for illustrative purposes, two of these QDs, labeled *i* and *j*. For the sake of clarity, we have assumed that each QD has radius $R_{QD}$. Their centers are separated by a Euclidean distance $d_{E}\left(i,j\right)$. As shown in Figure 3a, we have also represented an ξ axis that passes through the centers of both QDs. The ξ axis will assist us in clearly representing the associated concepts in Figure 3b,c. Figure 3b shows the corresponding finite QCP caused by a QD along ξ axis: $V\left(\xi \right)=-V_{C}$ (inside the QD) and V(ξ)=0 (otherwise). Since the QCP is finite, there is a part of the electron wavefunction that spreads outside the QD, as qualitatively shown in Figure 3c. Note that the QDs are close enough that the electron wavefunctions overlap. According to QM, the electron, is in both QDs with a probability amplitude as the one in Figure 3c. The electron can tunnel from one QD to the other. We model this quantum phenomenon by forming a link between nodes *i* and *j* (Figure 3d). We will show throughout the paper that the link weight $w_{ij}$ between two nodes *i* and *j* depends on the extent to which the electron wave-functions in both nodes overlap. Quite often, however, there are electron wave-functions in sufficiently remote QDs that do not overlap at all. Regarding this, we have represented in Figure 3e two nodes that are so far apart that their corresponding wave-functions do not overlap (Figure 3g). Thus, an electron that is in node *i* at the initial time t=0 will remain localized in that node even if t→∞. We model this QM result through the absence of a link ($w_{ij}=0$), as shown in Figure 3h (not connected nodes).

Our main result is that the proposed RGG is able to capture inner properties of the complex quantum system: it predicts the system quantum state, its time evolution, and the emergence of quantum transport (QT) as the QD density increases. In fact, QT efficiency exhibits an abrupt change, from electron localization (no QT) to delocalization (QT emerges). This is an electron localization–delocalization transition that has also observed in [60].

Our proposal could have potential application not only in improving the efficiency of QD-based optoelectronic devices (LEDs, solar cell, lasers, etc.) that make use of SAQD layers but also in single, huge macromolecules (light-harvesting molecules [61]) to study the quantum transport (energy, charge) between specific areas of their structures.

The rest of this paper has been structured as follows. After reviewing of the current state of the art in Section 2, Section 3 briefly introduces the QD system that we want to study, while Section 4 explains our RGG proposal. The experimental work in Section 5 allow for predicting inner features of the system such as the system quantum state, its time evolution, or the emergence of quantum transport. Section 6 discusses potential applications, strengths, and weaknesses of the proposed method. Finally, Section 7 completes the paper. Appendix A lists the symbols used in this work.

## 2. Current State of the Art

There are basically two approaches that combine CN and QM concepts [62]. The first one applies concepts inspired by QMs to better study CN. For instance, Ref. [63] proposes a way to navigate complex, classical (non-quantum) networks (such as, the Internet) based on quantum walks [64] (the quantum mechanical counterpart of classical random walks [65]). More examples belonging to this first framework can be found in [62] and in the references therein. The second approach, in which the present article can be included, is based on applying CN concepts to explore nanosystems, which are governed by the laws of QM [66] and not by those of classical physics. A representative instance is the system studied in [67]: any atom trapped in a cavity is represented by a node, while the photon that the two atoms (nodes) exchange is encoded by a link between them. This and other papers have in common the fact of studying quantum properties on networks using quantum walks. This is because the quantum dynamics of a discrete system can be re-expressed and interpreted as a single-particle quantum walk [68,69]. This is the reason why quantum walks have been used to study the transport of energy through biological complexes involved in light harvesting in photosynthesis [70]. Quantum walks have also been used to explore transport in systems described by means of CN with different topologies [71,72]. Specifically, continuous-time quantum walks (CTQW)—a class of quantum walks on continuous time and discrete space [64]—have been used extensively to study quantum transport (QT) on CN [72], and will also be used in our work. There are several works that have studied QT over regular lattices [72,73,74], branched structures [75,76] (including dendrimers [76]), fractal patterns [77], Husimi cacti [78], Cayley trees [79], Apollonian networks [80], scale-free networks [81], small-world (SW) networks [82] and start graphs [83,84], leading to the conclusion that QT differs from its classical counterpart. Having a quantitative measure of the efficiency of QT in a CN has been found to be important for practical and comparative purposes. In this regard, Ref. [85] has recently found bounds that allow for measuring the global transport efficiency of CN, defined by the time-averaged return probability of the quantum walker. QT efficiency can undergo abrupt changes, and can have transitions from localization (no QT) to delocalization (QT appears). In this respect, the authors of [60] have studied localization–delocalization transition of electron states in SW networks. The SW feature is interesting because it makes it easy to navigate a network since SW networks exhibit a relatively short path between any pair of nodes [86,87]: the mean topological distance or average path length *ℓ* is small when compared to the total number of nodes *N* in the network (ℓ=O(lnN) as N→∞). The usual technique of rewiring [86] or adding links [88] in macroscopic, non-quantum CN to create SW networks have also been extended to quantum system [82,84] to enhance QT. In [82], SW networks have been generated from a one-dimensional ring of *N* nodes by randomly introducing *B* additional links between them. The quantum particle dynamics has been modeled by CTQWs, computing the averaged transition probability to reach any node of the network from the initially excited one. Finally, the strategy of adding new links have been explored in star networks with the aim of enhancing the efficiency of quantum walks to navigate the network [84]. Please note that all of these key works have focused their research from the viewpoint of the topological properties. In particular, the topological (geodesic) distance between two nodes *i* and *j*, d(i,j), is the length of the shortest path (geodesic path) between them, that is, the minimum number of links when going from one node to the other [50]. The distance between two nodes *i* and *j* that are directly linked is d(i,j)= 1, regardless of where they are located in physical space. We propose in the next paragraph to use the Euclidean distance for reasons that will be clearer later on.

## 3. The QD System

Let us consider a microscopic physical system, which is closed, and made of a set of *N* QDs that are randomly distributed in a metric space as shown in Figure 2a. The position of each nanostructure in the metric space $R^{2}$ is determined by a position vector r. We consider a single electron (walker) freely tunneling among QDs (when allowed). An example of one-electron model is the tight-binding model [89], which has been used for both lattice and random networks.

Aiming to later numerically illustrate the results of studying this system using CN concepts, we are going to assume a set of hypotheses about the QCF that the QDs produce. These hypotheses will allow us to tackle the problem by using some well known QM results on each individual nanostructure, so that we will be able to then focus on exploring the complete system as a RGG.

**Figure 4:**
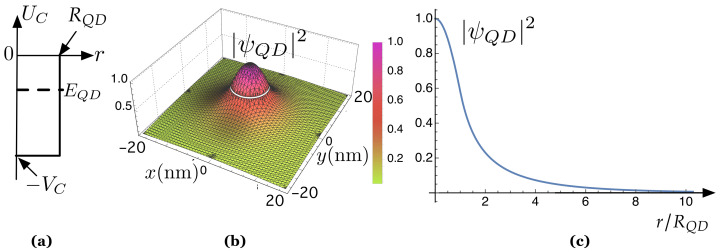
(**a**) Quantum confinement potential. $V_{C}$ is the depth of the potential well while $R_{QD}$ is the radius of the QD producing $U_{C}$. $E_{QD}$ is the energy of the bound state; (**b**) squared modulus of the corresponding wave function, $|\psi_{QD}{|}^{2}$, in Cartesian coordinates; (**c**) $|\psi_{QD}{|}^{2}$ as a function of the normalized radial coordinate, $r/R_{QD}$.

With this in mind, and for reasons that will be clearer later on, we first assume that the single band effective mass equation of electrons in the envelope approximation [90] is a proper description of the dot and barrier bulk materials. This is because a QD size of 10 to 20 nm is much larger than the lattice constant of the material involved and, thus, it seems reasonable to consider that only the envelope part of the electron wave function is affected by the confinement potential. For the sake of clarity, we also assume that the QDs are identical (since in a closed system, energy is conserved, and the electron can only make transitions between QDs that have the same energy, that is, between QDs that have the same size [91]) and spherical with radius $R_{QD}$. The center of any QD *i* is given by a position vector $r_{i}$ in the metric space. We assume that its associated QCP is spherically symmetric (depending only on the radial co-ordinate *r*), finite and “square”:

(1) $$U_{C}\left(r\right)=\left\{\begin{array}{cc} -V_{C} & ,if\;r<R_{QD}\\ 0 & ,if\;r>R_{QD} \end{array}\right.,$$

as shown in Figure 4a.

The reason why we have made use of $U_{C}$ is that the time-independent Schrödinger’s equation, which allows for computing both the electron wavefunction ($\psi_{QD}$) and its energy ($E_{QD}$),

(2) $$\hat{H}\psi_{QD}=E_{QD}\cdot \psi_{QD},$$

can be solved analytically [91,92,93]. $\hat{H}$ in Equation (2) is called the Hamiltonian operator and corresponds to the total energy of the system,

(3) $$\hat{H}\equiv -\frac{\hbar^{2}}{2m}{▿}^{2}+\hat{V}$$

where *ℏ* is the reduced Planck constant, *m* the electron mass, ${▿}^{2}$ is the the Laplace operator [94], and $\hat{V}$ is the energy potential operator.

Thus, according to QM, the electron energy in the QCF (1) is quantized: it can only take discrete values [91]. The number of “bound states” in this QCF depends on $V_{C}\cdot {\left(2R_{QCP}\right)}^{2}$ (see [91]): there is a range of values of $V_{C}\cdot {\left(2R_{QCP}\right)}^{2}$ for which there is only one energy level. Now, if we assume, for simplicity, that the QD size, $2R_{QCP}$, is so tiny that there is only one energy level, $E_{QD}$, its associated wavefunction is a 1 *s*–orbital [6,93]. We have solved the problem for a single, isolated QD with $R_{QCP}=10$ nm and $V_{C}=0.47$ eV, typical in III-V semiconductors. There is only one bound state, whose energy is $E_{QD}=0.4$ eV. Its associated wave function $\psi_{QD}$ is a spherical 1s−orbital. We have represented its squared modulus, $|\psi_{QD}{|}^{2}$, in both Cartesian (Figure 4b) and radial coordinates (Figure 4c). The latter shows how $|\psi_{QD}{\left(r\right)|}^{2}$ decreases very quickly as a function of the normalized radial coordinate, $r/R_{QCP}$.

With this idea in mind, the potential in the complete system is a function that varies from one QD to another, taking the $-V_{C}$ value inside each QD and zero in the space among QDs:

(4) $$V\equiv V\left(r\right)=\left\{\begin{array}{cc} -V_{C} & ,inside\;a\;QD\;at\;r\\ 0 & ,outside\;a\;QD, \end{array}\right.$$

where r is the position vector locating each QD.

## 4. Modeling the QD System as a Spatial Network: The Proposed Model

**Figure 5:**
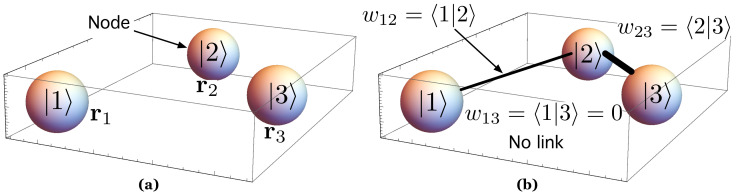
(**a**) System with three QDs. Vector r is the position vector in the metric space. We represent each QD as a node, and we label it with the ket notation |i〉; (**b**) methodology to form weighted links according to QMs. Each weight $w_{ij}$ is the overlap integral between $\psi_{QDi}$ and $\psi_{QDj}$ (or, equivalently, 〈i|j〉 in Dirac’s notation).

Aiming at generating the network associated with the proposed system, we represent any QD *i* by means of a *node*, and we label this node using its ket |i〉, as shown in Figure 5a. Our next step is to generate the links in a way that makes physical sense according to QM, and also takes into account that the nodes are spatially embedded. As outlined in Section 1 when introducing our approach, we generate a link between two nodes (sites, kets), |i〉 and |j〉, located at $r_{i}$ and $r_{j}$, respectively, by computing to what extent their respective wave functions overlap. We compute the overlap between the wave functions $\psi_{QD_{i}}$ and $\psi_{QD_{j}}$ as the overlap integral [94] over all the physical space *S*

(5) $$\int_{S}\psi_{QD_{j}}^{*}\psi_{QD_{i}}\;dr=\langle j|i\rangle =w_{ij}.$$

Note that, because of symmetry, 〈j|i〉=〈i|j〉.

**Figure 6:**
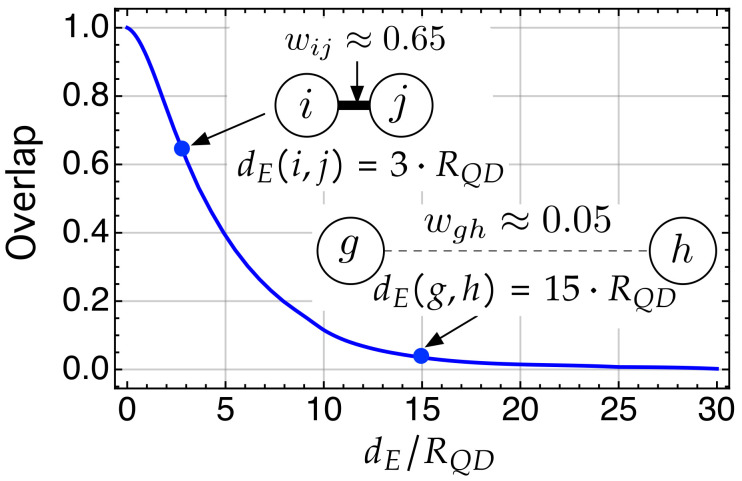
Overlap between the electron wave functions in two QDs as a function of the distance (between dot centers) normalized by the radius of the QD, $d_{E}/R_{QD}$. The insets aim to graphically illustrate how increasing the separation between the nodes implies a longer link with much less weight.

Using Expression (5), we have computed the overlap integral for nodes whose centers are separated by a *normalized* Euclidean distance $d_{E}/R_{QCP}$. The result appears in Figure 6, where we have represented the overlap integral as a function of $d_{E}/R_{QCP}$. We have highlighted two situations in Figure 6 for illustrative purposes.

The first one corresponds to the inset in which the centers of the nodes (sites) *i* and *j* are separated by a distance $d_{E}\left(i,j\right)=3\cdot R_{QD}$ for which $\langle i|j\rangle =w_{ij}\approx 0.65$. Thus, we generate a link between nodes *i* and *j* whose weight is $w_{ij}=0.65$. The second inset corresponds to two nodes whose centers are at a $d_{E}\left(g,h\right)=15\cdot R_{QD}$ for which $w_{gh}=\langle g|h\rangle =w_{gf}\approx 0.05$. A link is generated, but has a very small weight $w_{gh}\approx 0.05$. Note that the overlap integral $w_{ij}\to 0$ when $d_{E}\left(i,j\right)>25\cdot R_{QD}$, and is $w_{ij}=0$ for $d_{E}\left(i,j\right)\geq 30\cdot R_{QD}$. That is, in our system, all those nodes whose centers are separated by a distance $d_{E}\geq 30\cdot R_{QD}=d_{E,Lim}$ (or *limit* distance) are *not allowed to be linked*. This is the case of nodes |1〉 and |3〉 in Figure 5, where $\langle 1|3\rangle =w_{13}=0$.

Although it does not seem clear yet at this point,

(6) $$d_{E,Lim}\equiv d_{S},$$

defines a new *scale* in the system, $d_{S}$. Note that our approach is a modified version of a RGG. As mentioned, an RGG is the simplest spatial network, consisting of randomly placing *N* nodes in some metric space and connecting two nodes by a link if and only if their Euclidean distance is smaller than a given neighborhood radius, *r*. In our case, this is distance is $d_{E,Lim}$. The novelty of our approach is that any link between nodes *i* and *j* is characterized by a weight that involves the overlap integral between kets |i〉 and |j〉.

To advance in our model, it is necessary to introduce some essential concepts. The first one arises from the very interaction between nodes. When two nodes are directly connected by a link, they are then said to be “adjacent” or neighboring. The adjacency matrix A encodes the topology of a network, that is, whether or not there is a link ($a_{ij}=1$ or $a_{ij}=0$) between any two pairs of nodes *i* and *j*. Sometimes, this binary information encoding whether or not a node is connected to another is not enough, and it is necessary to quantify the “importance” of any link (the strength of a tie between two users in a social network, or the flow of electricity between two nodes in a power grid [35]) by assigning to each link a “weight”. In that case, the matrix that encodes the connections is called weighted adjacency matrix W [41]. With our method, the *weighted adjacency matrix* corresponding to the network represented in Figure 5b is

(7) $$\left(\begin{array}{ccc} 0 & \langle 1|2\rangle & \langle 1|3\rangle \\ \langle 2|1\rangle & 0 & \langle 2|3\rangle \\ \langle 3|1\rangle & \langle 3|2\rangle & 0 \end{array}\right)=\left(\begin{array}{ccc} 0 & \langle 1|2\rangle & 0\\ \langle 2|1\rangle & 0 & \langle 2|3\rangle \\ 0 & \langle 3|2\rangle & 0 \end{array}\right),$$

which is symmetric (since 〈i|j〉=〈j|i〉 in this quantum systems), off-diagonal and non-negative. In particular, 〈1|3〉=〈3|1〉=0 since there is no overlap between the electron wave functions in kets |1〉 and |3〉.

An interesting point in Expression (7) is that its matrix elements have in QM the meaning of *probability amplitude*, 〈i|j〉, which is related to the *probability* for an electron to be in |i〉 and |j〉, $P_{ij}$, as follows:

(8) $${\langle j|i\rangle }^{*}\langle j|i\rangle ={\left|\langle j|i\rangle \right|}^{2}.$$

We can now generalize the idea from the toy system in Figure 5 to the complete, complex, quantum system composed of *N* QDs that are independently and uniformly distributed in the metric space $R^{2}$. The corresponding adjacency matrix is thus an N×N*weighted adjacency matrix*$W_{PA}$ whose matrix elements, 〈i|j〉, are the *probability amplitude* for an electron in kets |i〉 and |j〉,

(9) $${\left(W_{PA}\right)}_{ij}=\left\{\begin{array}{cc} 0 & ,if\;i=i\\ \langle i|j\rangle & ,if\;i\neq j \end{array}\right.$$

Once we have defined our weighted adjacency matrix, $W_{PA}$ in (9) and interpreted its meaning in QM, we now have enough knowledge to represent the system as a network by using the undirected, weighted graph $G\equiv G(N,L,W_{PA})$, where N is the set of nodes (card(N)=N) and L is the set of links. We have specified the matrix $W_{PA}$ in the triplet $G\equiv G(N,L,W_{PA})$ to emphasize the fact that the connections between the nodes are made using the $W_{PA}$ matrix and not, for example, a conventional adjacency matrix A ($a_{ij}=1$ if *i* and *j* are directly linked; 0 otherwise), which would result in different results.

Note that, because of the way we have generated the links, the weighted adjacency matrix $W_{PA}$ *quantifies* connections that have physical meaning according to QM, and explicitly includes the spatial structure of the system (remember Figure 6 and its associated, previous discussion). This is the key point that allows us to apply to $W_{PA}$ techniques that are well known in network science. For instance, in addition to the weighted adjacency matrix, it is common to use the diagonal degree matrix D, whose elements $D_{i}$ are the sum of weights of all links directly connecting node *i* with others. In our particular system, D has physical meaning: using (9), $D_{i}=\sum_{i\neq j}{\left(W_{PA}\right)}_{i}=\sum_{i\neq j}\langle i|j\rangle$$\equiv s_{AP_{i}}$ is the sum of the probability amplitudes on ket |i〉. We label it $s_{AP_{i}}$ to stress this physical meaning.

$W_{PA}$ helps us obtain Laplacian matrices that will assist us in studying electron dynamics using CTQW, quantum walks that are continuous in time and discrete on space. See [64] for a very illustrative discussion on CTQW and their use in the simulation of quantum systems. The first type of Laplacian matrix, the (combinatorial) Laplacian, or simply, Laplacian matrix,

(10) $$L=D-W_{PA}.$$

Note that the Laplacian matrix L computed using the weighted adjacency matrix $W_{PA}$ is different from the one used in other works [72,84,95]. In these approaches, the matrix elements of L are assumed to be equal $\gamma_{ij}\equiv \gamma =1$. In our approach, the matrix elements take different values since they depend on the involved overlap integrals (or probability amplitudes, $0\leq w_{ij}<1$) and, as shown throughout the paper, they play a natural role in the probability for an electron to tunnel from one node to another. The Laplacian acts as a node to node transition matrix. The Hamiltonian of the CTQW can be written as H=L [68,75,77,82,84,85,96,97,98,99,100,101].

The second one is the *normalized Laplacian matrix* [96], $L_{N}=$$D^{-1/2}LD^{-1/2}$, an Hermitian operator that, according to the way we have defined $W_{PA}$ in (9), has matrix elements in the form

(11) $${\left(L_{N}\right)}_{ij}=\left\{\begin{array}{cc} 1 & ,if\;i=j\\ -\frac{\langle i|j\rangle }{\sqrt{s_{APi}}\cdot \sqrt{s_{APj}}} & ,if\;i\neq j \end{array}\right.$$

$L_{N}$ allows for generating the corresponding unitary CTQW [96] of an electron on our graph $G\equiv G(N,L,W_{PA})$ as

(12) $${\hat{U}}_{L_{N}}\left(t\right)=e^{-iL_{N}\cdot t}$$

Note that, in the time evolution operator generated by $L_{N}$ in (12), the imaginary unit makes ${\hat{U}}_{L_{N}}$ be unitary [75]. As in other CN approaches [74,76,102,103], we assume ℏ≡1 so that time and energy can be treated as dimensionless. We will use ${\hat{U}}_{L_{N}}\left(t\right)$ to study the temporal evolution of our quantum system.

## 5. Experimental Work: Simulations

### 5.1. Network Parameters

The spatial constraints stated by the overlap integral have effects on CN parameters such as degree distribution, clustering, and average shortest path, defined as:

- The degree distribution of a network captures the probability P(k) that a randomly chosen node exhibits “degree” *k* (= number of links). P(k) and its mean value 〈k〉 (mean degree) are very useful since it quantifies to what extent nodes are heterogeneous with respect to their connectivity. In fact, many real-world networks exhibit broad, heterogeneous degree distributions. In a degree-heterogeneous network, the probability to find a node with k>〈k〉 decreases slower than exponentially, leading to the existence of a non-negligible number of nodes with very high degrees. A key feature of such degree distributions is the so-called scale-free behavior [32], characterized by a degree distribution $P\left(k\right)∼k^{-\gamma }$. This means that most of the nodes have very few links, while only a few nodes have a large percentage of all links. These most connected nodes are called “hubs”.
- The clustering or transitivity [32] quantify the probability that two neighbors of a given node *i* are connected. This concept is clear in social networks: the fact that usually “the friend of a friend is a friend” leads to high clustering coefficient. The “clustering coefficient” is a local property capturing “the density” of triangles in the graph, that is, two nodes that both are connected to a third node are also directly connected to each other. A node *i* in the network has $k_{i}$ links that connects it to $k_{i}$ other nodes. The clustering coefficient of node *i* is defined as the ratio between the number $M_{i}$ of links that actually exist between these $k_{i}$ nodes and the maximum possible number of links, that is, $C_{i}=2M_{i}/k_{i}\left(k_{i}-1\right)$. The clustering coefficient of the whole network is:
  (13) $$\langle C\rangle =\frac{1}{N}\sum_{i}C_{i}.$$
- The average shortest path length, *ℓ*, quantifies the extent to which a node is accessible from any other [32]. The average path length of a network is the average value of distances between any pair of nodes in the network:
  (14) $$\ell =\frac{1}{N(N-1)}\sum_{i\neq j}d_{ij}$$
  where $d_{ij}$ is the distance between node *i* and node *j*. The length of the shortest path between two nodes *i* and *j* in a network is the minimum number of links for going from node *i* to *j*. Its average is computed over all possible pairs of nodes. When *ℓ* is small when compared to the “network size” (number of nodes, *N*), the small-world property arises. Intuitively, this means that any pair of nodes are relatively “close”. Mathematically, this means that the average shortest path scales logarithmically with the network size [32]: ℓ∼lnN.

The fact that an electron cannot tunnel from one QD to another that is at a distance longer than $d_{E,Lim}$ leads to inhibiting the existence of long-range links to connect hubs (that is, shortcuts are not allowed). The increasing tendency to establish connections in the neighborhood ($d<d_{E,Lim}$) leads to a high clustering coefficient (〈C〉≈0.57).

### 5.2. The Network Has a Percolation Transition as the Dot Density Increases

Consider the density of QDs as $\rho_{QD}=N/A$, where A=1 is the area of a unit square. When $\rho_{QD}$ is very small (and, so does 〈k〉, *k* being the number of links per node), the nodes are so far apart that there is no overlap between the wave functions and, as a consequence, there is no links: 〈k〉→0 and there are *N* isolated nodes.

As $\rho_{QD}$ increases (and, so does 〈k〉), the QDs are closer and closer and electron wavefunctions in some of them start to overlap, causing the formation of links among some of them. As a consequence, small clusters begin to appear. They are disconnected from each other. These isolated subnetworks are called components and all of them have similar size.

**Figure 7:**
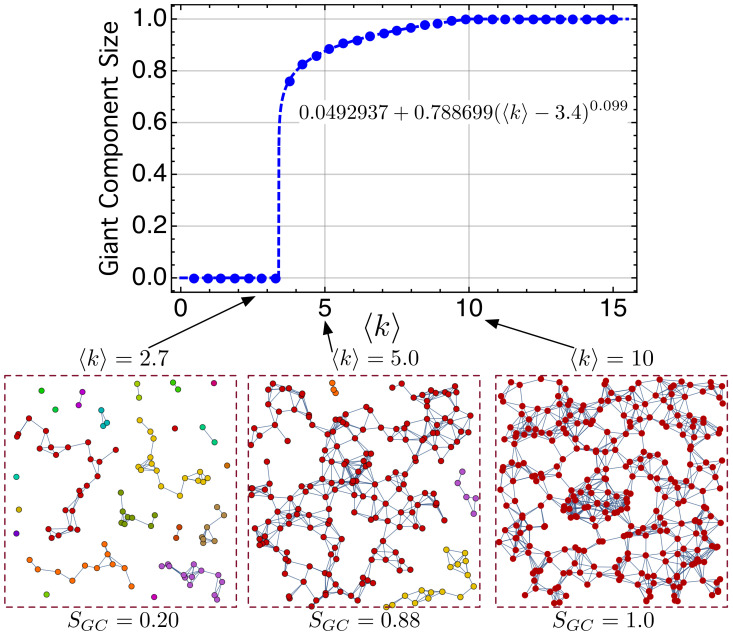
Normalized size of the giant component with respect to the total number of nodes, $S_{GC}=N_{GC}/N$, as a function of the average node degree 〈k〉. See the main text for further details.

However, there is a value of 〈k〉 for which one of the clusters becomes dominant and begins to grow to the detriment of the others. This cluster is called a giant component (GC). The fraction or normalized size of the giant component with respect to the total number of nodes is quantified as $S_{GC}=N_{GC}/N$. Figure 7 shows $S_{GC}$ of our network as a function of the average node degree 〈k〉. Note that, for 〈k〉<3.4, there is no giant component. There are several disconnected networks, many of then being trees. The point 〈k〉=3.4 in Figure 7 seems to be a critical point at which $S_{GC}$ has an abrupt transition. For 3.4<〈k〉<10, there is a single giant component with small clusters. For 〈k〉≥10, there is only a single giant component, that is, the network is connected.

This formation of a GC on a macroscopic scale is an example of percolation transition [104]. The fraction of nodes belonging to the giant cluster becomes the order parameter of the percolation transition. Following [104], we have denoted it as m(〈k〉). Ref. [104] explores the most recent advances of percolation theory in CN, and studies the order parameter *m* for continuous, explosive, discontinuous, and hybrid percolation transitions. According to [104], our network exhibits a Hybrid Percolation Transition (HPT) since it has properties of both second-order (critical phenomena) and first-order (abrupt jump of the order parameter) phase transitions at the same transition point, ${\langle k\rangle }_{C}$, in our case.

In our case, $m\left(\langle k\rangle \right)\equiv S_{GC}\left(\langle k\rangle \right)$ fulfills

(15) $$m\left(\langle k\rangle \right)=\left\{\begin{array}{cc} 0 & ,if\;\langle k\rangle <{\langle k\rangle }_{C}\\ m_{0}+r\cdot {\left(\langle k\rangle -{\langle k\rangle }_{C}\right)}^{\beta_{m}} & ,if\;\langle k\rangle \geq {\langle k\rangle }_{C} \end{array}\right.$$

where $m_{0}$ and *r* are constants, and $\beta_{m}$ is the critical exponent of the order parameter. For ${\langle k\rangle }_{C}\geq 3.4$, $m\left(\langle k\rangle \right)=0.0492937+0.788699{\left(\langle k\rangle -3.4\right)}^{0.099}$.

### 5.3. Studying the Emergence of Electron Transport

We characterize the network’s transport efficiency by using the *average return probability* $\bar{\alpha }\left(t\right)$, defined as [85]

(16) $$\bar{\alpha }\left(t\right)=\frac{1}{N}\sum_{j=1}^{N}{\left|\langle j|\hat{U}{,}_{L_{N}}\left(t\right)|j\rangle \right|}^{2},$$

where the operator ${\hat{U}}_{L_{N}}\left(t\right)$, stated in Equation (12), is the unitary time evolution operator governing the evolution of the probability amplitudes. Please note that, as shown in a number of papers [68,75,77,82,84,85,96,97,98,99,100,101], the Hamiltonian of the network is the Laplacian matrix (also called Connectivity matrix in some contexts).

High values of $\bar{\alpha }\left(t\right)$ suggest inefficient transport since the quantum particle tends to remain at the initial node [85]. On the contrary, $\bar{\alpha }\left(t\right)\ll 1$ means that the electron, localized at the initial node in t=0, tends to be delocalized, with different probability components on each node.

With this concepts in mind, we define the quantum transport efficiency as

(17) $$\eta_{QT}\left(t\right)=1-\bar{\alpha }\left(t\right).$$

**Figure 8:**
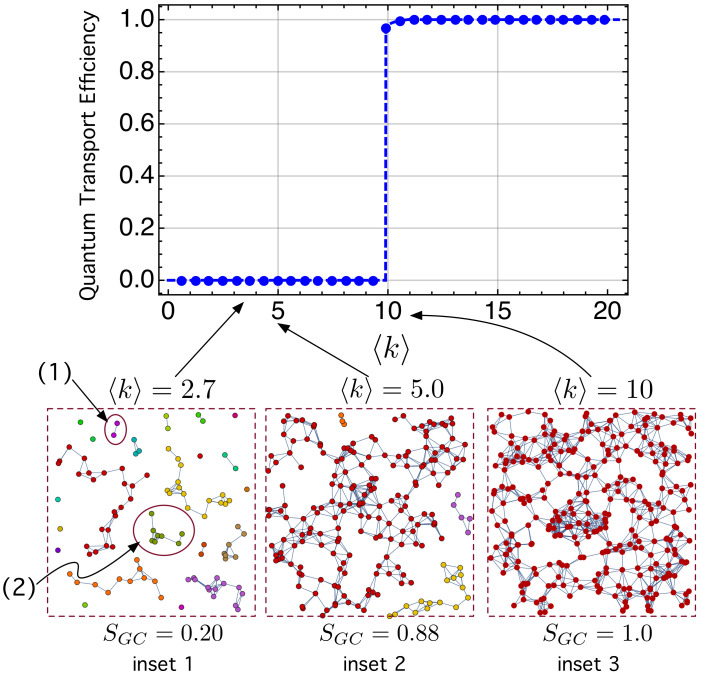
Quantum transport efficiency as a function of 〈k〉. Insets “1”, “2” and “3” shows network connectivity at different values of 〈k〉. $S_{GC}=N_{GC}/N$ is the fraction (normalized size) of the giant component.

Figure 8 shows the quantum transport efficiency $\eta_{QT}$ stated by (17) as a function of the average node degree 〈k〉, *k* being the number of links per node. Note that $\eta_{QT}=0$ for 〈k〉<10 while there is and abrupt transition at 〈k〉=10 so that $\eta_{QT}\approx 1$ for 〈k〉>10.

The results shown in Figure 8 has been computed with t=500. Note that time and energy can be treated as *dimensionless* when assuming ℏ≡1, as mentioned before [74,76,102,103].

To explain this result, we have studied what happens to the connectivity of the network when the density of QDs per unit area (*A*), $\rho_{QD}=N/A$, increases. When $\rho_{QD}$ is very small (and, so does 〈k〉), the nodes are so far apart that there is no overlap between wavefunctions and, as a consequence, there is no links: 〈k〉→0, and there are *N* isolated nodes. As $\rho_{QD}$ increases (and so does 〈k〉), the QDs are closer and closer and the electron wavefunctions in some of them start to overlap, causing the formation of links among some of them. This is the case of the two nodes labeled “(1)” in Figure 8. Small clusters begin to appear too (cluster labeled “(2)”). Note that they are disconnected from each other. These isolated subnetworks are called components and many of them have a similar size. In any component, the electron can tunnel among the involved QDs. This is just the situation in the below, leftmost inset, called “inset 1”.

However, as 〈k〉 continues to grow, one of the clusters becomes dominant and begins to grow more and more as forming links to other smaller clusters. This cluster is called a giant component (GC). The fraction or normalized size of the giant component with respect to the total number of nodes is quantified as $S_{GC}=N_{GC}/N$. In “inset 2”, with 〈k〉=5, we can see that there are small clusters along with a single GC whose size is $S_{GC}=0.88$.

**Figure 9:**
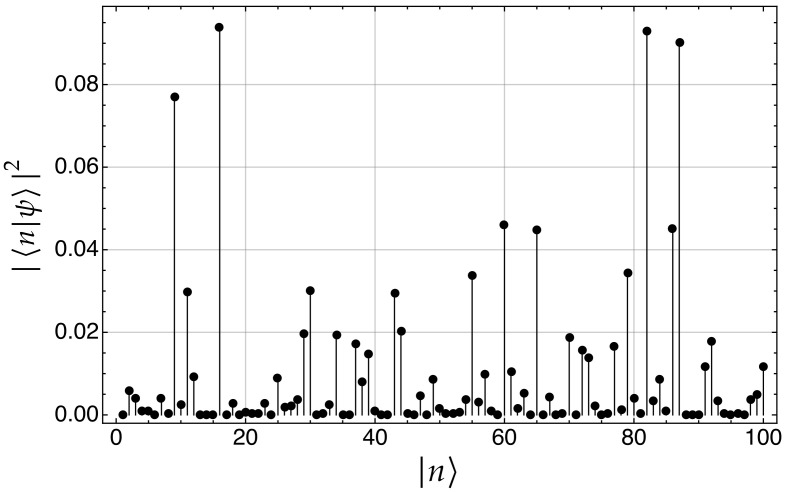
Electron probability components, ${\left|\langle n|\psi \rangle \right|}^{2}$, on each of the kets |n〉 of a connected network with N=100 nodes.

Just when 〈k〉 increases up to 〈k〉=10, one single GC ($S_{GC}=1$) appears, which has captured all the other small components, making the complete network be *connected*. The electron can thus tunnel from any node to any other node. When the network is connected, an electron always has at least one path to pass from any node *j* to another. As a consequence, $\bar{\alpha }\left(t\right)=0$ in Equation (16) since the electron is no longer confined at node *j*, and can now behave as an *extended* wave function whose components are distributed (although with different probability components) between different nodes (see Figure 9). As $\bar{\alpha }\left(t\right)\approx 0$, then the quantum transport efficiency (17) is $\eta_{QT}\left(t\right)=1-\bar{\alpha }\left(t\right)\approx 1$.

Figure 9 is an example of the electron probability components, ${\left|\langle n|\psi \rangle \right|}^{2}$, on each of the kets |n〉 of a network with N=100 nodes. In this example, the electron was localized in node |7〉 at t=0 (initial state), and after applying the unitary evolution operator (12) for t=500, it has evolved to a extended wave function whose probability components ${\left|\langle n|\psi \rangle \right|}^{2}$ are distributed over the N=100 nodes.

For the sake of clarity, Table 1 lists the corresponding numerical values represented in Figure 9. Please note that the sum of the probability components in the whole system is $\sum_{n=1}^{N=100}{\left|\langle n|\psi \rangle \right|}^{2}=1$.

## 6. Potential Applications, Strengths, and Weaknesses of the Proposed Method

### 6.1. Prospective Applications

To the best of our knowledge, there are two possible groups of applications: intermediate band (IB) materials and light-harvesting materials.

#### 6.1.1. Intermediate Band Materials

The first potential application of the proposed method could be found within the field of IB materials to put into practice the concept of intermediate band solar cell (IBSC) [105]. This solar cell has an isolated IB within what, otherwise, would be the semiconductor gap, $E_{G}$. The IBSC was proposed by Luque and Martí in [106] and exhibits a limiting efficiency of 63.2%, much higher than the Shockley–Queisser (SQ) limit [107] of the single-gap solar cell (40.7%). Unlike other sub-bandgap absorbing proposals, the IBSC concept surpasses the SQ limit by means of: (1) increasing the photogenerated current via the two-step absorption of sub-bangap photons via an IB located within the gap $E_{G}$; and (2) without degrading the cell output voltage. Several technological approaches have been proposed aiming to obtain IB materials. The first one, the QD approach has led to the quantum dot intermediate band solar cell (QD-IBSC) [108], the first device on which it has been possible to experimentally prove the two concepts the IBSC is based on [109,110,111,112]. A sufficiently dense array of QDs with a single bound state leads to an IB material. Regarding this, a possible future application of our method could consist in exploring the electron conductivity in the IB that arises from the bound states within the QDs. A second feasible strategy for obtaining IB materials is based on semiconductor bulk materials containing a high density of adequate deep-level impurities and those corresponding to materials that “naturally” have an IB (theoretically predicted by ab-initio methods) [113,114,115,116,117,118,119]. Regarding the use of deep-level impurities, the proposed method could potentially be used to study when a sufficiently large density of impurities produces a transition from localized states (in the impurities) to states that are extended to the whole volume of the host semiconductor. It could be interesting to compare with experimental data [120,121] and theoretical models [122]. Putting it simply, deep centers transform into a band when their density is sufficiently high (Mott transition) [123,124], and, as a consequence, the electron wave-function becomes extended instead of localized. Regarding this, the experimental test of the theoretical model stated in [122] have been recently proved in silicon implanted with Ti [120] and in silicon supersaturated with sulfur [121].

#### 6.1.2. Light-Harvesting Materials

In the field of light-harvesting materials [125], QDs have been found to be promising light-harvesting materials because of their size-, shape-, and composition-dependent electronic properties, and exciton generation after photoexcitation [126]. The key challenge is to model and design nanoscale materials with tailored properties for light harvesting work. Regarding this, our proposal could be used to explore the transport of energy (excitons) in light-harvesting QDs [126], light-harvesting molecules [125,127,128], polymer nanoparticles [129], dimers, and networks [130].

### 6.2. Strengths and Weaknesses

The proposed method has a set of strengths and weaknesses. Among the strengths, the method allows for obtaining more realistic simulations of nanostructures than others found in the literature, where the fact that the tunneling probability decreases exponentially with the distance is not considered. The method is also generalizable to nanostructures other than QDs, such as impurities in semiconductors, light-harvesting molecules, etc. Another advantage is that it allows obtaining an approximation of the electron behavior in systems with a high number of components per area unit (whether they are QDs or impurities). The main weakness of the proposed method is that it is a first approach in the sense that identical quantum dots have been assumed. However, self-assembled quantum dot growth methods produce ensembles of quantum dots that have size dispersion, which leads to a dispersion in bound levels from one QD to another. The probability of electron hopping from a QD to another with the same energy level must be considered when computing the weight of links. Additionally, it is necessary to consider not only the electron system but also the phonon system, which provides/absorbs the energy when an electron hops from one bound state in a QD to another with different energy. Because of its complexity, we leave this refinement for future work.

## 7. Summary and Conclusions

This paper has proposed the use of a special class of Random Geometric Graphs (RGG) to model systems formed by *N* disordered quantum dots (QDs). While discerning what a node is seems easy (QD ≡ node), what requires a bit more care and physical intuition is determining how the links between QDs are formed in such a way that they have meaning. Specifically, in the network model that we have proposed, the most novel aspect is the link formation mechanism between nodes (≡ QDs): any link between two nodes *i* and *j* is formed if and only if the corresponding electron wave function at such nodes have non-zero overlap. Any link has thus a weight $w_{ij}$ that is the real number ($0\leq w_{ij}<1$) corresponding to the electron overlap integral (also called *probability amplitude* (PA)).

The aforementioned link formation mechanism leads to a N×N*weighted adjacency matrix*$W_{PA}$ whose matrix elements are the *probability amplitudes* for a single electron in the involved nodes. The corresponding Laplacian matrix L, which assists in computing continuous time quantum walks (CTQW) on the associated network, is *different* from the one used in other works [72,84,95]. In these approaches, the matrix elements of L are assumed to be equal $\gamma_{ij}\equiv \gamma =1$. In our approach, the matrix elements take different values since they depend on the involved overlap integrals (or probability amplitudes, $0\leq w_{ij}<1$) and, as shown throughout the paper, they play a natural role in the probability for an electron to tunnel from one node to another.

Regarding this, our main results point out:

1. The spatial network generated by the proposed model prohibits the existence of shortcuts between distant nodes because of the impossibility of the electron tunneling between two very distant QDs. This leads, as expected, to high clustering coefficient and makes it impossible for the network to be small-world.
2. The proposed network is also able to capture the inner properties of the QD system: it predicts the system quantum state, its time evolution, and the emergence of quantum transport (QT) as the mean node degree increases (or, equivalently, when the QD increases). In fact, QT efficiency exhibits an abrupt change, from electron localization (no QT) to delocalization (QT emerges), which has also been observed in [60], although with a different approach.

## Figures and Tables

**Table 1:** List of the electron probability components, ${\left|\langle n|\psi \rangle \right|}^{2}$, on each of the kets |n〉 of a connected network with N=100 nodes. The sum of the probability components in the whole system is $\sum_{n=1}^{N=100}{\left|\langle n|\psi \rangle \right|}^{2}=1$.

0.000172176	0.00582029	0.00387988	0.00106017	0.000970012
0.0000719148	0.00413783	0.000454207	0.0768675	0.00242522
0.0298138	0.00921407	0.0000565646	0.000102786	0.0000761972
0.0938865	0.00010824	0.0027705	0.0000545876	0.000735455
0.000247052	0.000190905	0.00274959	0.0000908889	0.00886395
0.00197876	0.00202883	0.003812	0.0197223	0.0300932
0.000148517	0.000360844	0.00247567	0.0193588	0.0000203756
0.0000281	0.0172012	0.00814919	0.0147341	0.00103512
0.000150275	0.0000862307	0.0294795	0.0203633	0.00025177
0.0000518012	0.00469129	0.0000315912	0.00847614	0.00144027
0.000285581	0.000261477	0.000582733	0.00371945	0.0336232
0.0032206	0.00980407	0.000807111	0.000121867	0.0459497
0.0105552	0.00168941	0.00534086	0.000115002	0.0447992
0.0001218	0.00431284	$8.72451\times 10^{-6}$	0.000201637	0.0188286
0.000133976	0.0156714	0.0137177	0.0022036	0.000168711
0.000250133	0.0166772	0.00114926	0.0344186	0.00393284
0.000493777	0.0928017	0.00341317	0.00876622	0.000893781
0.0451846	0.0901622	0.0000291204	0.0000476467	0.0000574528
0.0117769	0.0178166	0.0034605	0.000244178	0.000142244
0.00037752	0.000129045	0.00373535	0.00499453	0.0118115

## Abbreviations

The following abbreviations are used in this manuscript:

0D	Zero-dimensional
1D	One-dimensional
2D	Two-dimensional
CN	Complex Networks
CTQW	Continuous-Time Quantum Walks
GC	Giant Component
IB	Intermediate Band
IBSC	Intermediate Band Solar Cell
QCP	Quantum Confinement Potential
QD	Quantum Dot
QD-IBSC	Quantum Dot Intermediate Band Solar Cell
QM	Quantum Mechanics
QT	Quantum Transport
RGG	Random Geometric Graph
RN	Random Network
SAQDs	Self-Assembled Quantum Dots
SML-QDs	Sub-monolayer Quantum Dots
SN	Spatial Network
SK	Stranski–Krastanow
SW	Small-world
VW	Volmer–Weber
WL	Wetting layer

## Appendix A

A	Adjacency matrix of a graph G.
$a_{ij}$	Element of the adjacency matrix A
$\bar{\alpha }\left(t\right)$	Average return probability
〈C〉	Mean clustering coefficient of a network.
D	Node degree matrix: diag $\left(k_{1},\cdots ,k_{N}\right)$. It is the diagonal matrix formed from the nodes degrees.
$d_{E}\left(i,j\right)$	Euclidean distance between any pair of nodes *i* and *j* in a network.
$d_{ij}$	Distance between two nodes *i* and *j*. It is the length of the shortest path (geodesic path) between them, that is, the minimum number of links when going from one node to the other.
$d_{E,Lim}$	$d_{E,Lim}\equiv d_{S}$ Euclidean distance limit beyond which there is no link formation.
$E_{QD}$	Discrete electron energy in a quantum dot (QD).
$\eta_{QT}$	Quantum transport efficiency.
G	Graph $G\equiv G(N,L,W_{PA})$, where N is the set of nodes (card(N)=N), L is the set of links, and $W_{PA}$ is weighted adjacency matrix that emerges from our method to link formation.
$\hat{H}$	Hamiltonian operator corresponding to the total energy of a quantum system.
H	Hamiltonian in matrix form.
*h*	Planck constant.
*ℏ*	Reduced Planck constant.
|i〉	Ket vector in the Hilbert space H. It corresponds to the electron wave function in nanostructure (≡ site ≡ node ≡ ket) *i*.
〈i|	Bra vector in the *dual space* corresponding to the ket |i〉 ∈H
〈k〉	Average node degree.
$k_{i}$	Degree of a node *i*. It is the number of links connecting *i* to any other node.
*ℓ*	Average path length of a network. It is the mean value of distances between any pair of nodes in the network.
L	Set of links (edges) of a network (graph).
L	Laplacian matrix of a graph G.
$L_{N}$	Normalized Laplacian matrix, $L_{N}=$ $D^{-1/2}LD^{-1/2}$.
*m*	Electron mass.
*M*	Size of a graph G. It is the number of links in the set L.
*N*	Order of a graph G=(N,L). It is the number of nodes in set N, that is, the cardinality of set N: $N=\left|N\right|\equiv \mathrm{card}\left(N\right)$.
N	Set of nodes (or vertices) of a graph.
${▿}^{2}$	Laplace operator.
$P_{j↝k}$	Probability for an electron to evolve between kets |j〉 and |k〉 in the time interval *t*.
P(k)	Probability density function giving the probability that a randomly selected node has *k* links.
|ψ〉	Ket or vector state in Dirac notation corresponding to the wave function ψ.
$R_{QD}$	Radius of the quantum dot.
$\psi_{QD}$	Electron wavefunction in a quantum dot.
$S_{GC}$	$S_{GC}=N_{GC}/N$ normalized size of the giant component (GC) with respect to the total number of nodes *N*.
$s_{AP_{i}}$	Sum of the probability amplitudes on ket |i〉, $s_{AP_{i}}\equiv$ $\sum_{i\neq j}{\left(W_{PA}\right)}_{i}=\sum_{i\neq j}\langle i|j\rangle$.
$\hat{V}$	Potential energy operator.
$-V_{C}$	Depth of confinement potential.
$U_{C}\left(r\right)$	Confining, spherical (depending only on the radial co-ordinate *r*), *finite*, and “square” potential energy.
${\hat{U}}_{L_{N}}\left(t\right)$	Time evolution operator generated by the normalized Laplacian matrix $L_{N}$.
$w_{ij}$	Weight of the link between node *i* and *j*. We define it as the overlap integral between the electron wave functions in kets *i* and *j* or the probability amplitude 〈i|j〉.
$W_{PA}$	weighted adjacency matrix whose elements are quantum probability amplitudes.
